# Reconstruction and the Voluntary Hospitals

**Published:** 1918-12-21

**Authors:** G. Q. Roberts

**Affiliations:** Secretary St. Thomas' Hospital.


### RECONSTRUCTION AND THE VOLUNTARY HOSPITALS.

By G. Q. ROBERTS, M.A., Secretary St. Thomas' Hospital.

Possibly the greatest election in the history of the Parliament of this country is taking place at the present time. The electors are being rallied round the cry of reconstruction. Candidates and voters alike are aiming at the one object—that the mass of the people shall, after this great war, live under conditions better than those they had attained through many years of struggle in this great democracy of ours.

Of the measures of reconstruction the most important will be those which aim at securing the health and happiness of the people. On November 7 last a Bill "to establish a Ministry of Health and a Board of Health to exercise in England and Wales and Scotland respectively powers with respect to health and local government and for purposes connected therewith" was introduced. It is to be the duty of the Minister to take all such steps as may be desirable to secure the effective carrying out and co-ordination of measures conducive to the health of the people, including measures for the prevention and cure of diseases, the treatment of mental and physical defects, the collection and preparation of information and statistics relating thereto, and the training of persons engaged in health services.

### Two Alternatives.

As fully as this brief article will allow, an indication will be given of the work hitherto devolving on the voluntary hospitals, so that our readers may consider how far the powers and duties of the Ministry in relation to health will affect their future. All who are familiar with the work which has been carried out by the voluntary hospitals of England feel strongly, in the words of a resolution recently passed by the British Hospitals Association, "That the interests of the patients and of the community as a whole, the progressive education and training of doctors and nurses, and the prosecution and advance of scientific research will be best served and provided for by the retention of the voluntary hospital system." This system is founded on the truest spirit of Christianity, the spirit of charity, and the alternatives between which we have to choose in the future are therefore :

1. The retention of the voluntary hospital system, or
2. The transference of their work to the control of the State.

Should the latter alternative ever be adopted we shall have clearly a State Medical Service somewhat analogous to either the Royal Army Medical Service, the Royal Naval Medical Service, or the present Poor-Law administration. All these services have existed side by side with the voluntary system, but it is principally to the voluntary system that England has owed most progress in medicine and surgery during the whole period of the growth of our social system, more especially during the last two centuries. When our monastic institutions were swept away by the Great Reformation it was recognised that provision must be made for the treatment of the sick and needy. The two ancient hospitals, St. Bartholomew's and St. Thomas's, were claimed by the citizens of London, and refounded as institutions entirely apart from political and municipal control. The great men who undertook the government of these institutions were public men, but it was as individuals that they gave their services, of inestimable value because of their ability and experience, to this great charitable work. As London grew and as new centres of population were formed throughout Great Britain, other hospitals of precisely the same character were established.

By the middle of the eighteenth century the earliest Schools of Medicine was founded, in close connection with the greatest of these hospitals. Such a plan was thoroughly typical of the British character and its spirit of independence. The healthy rivalry created led to rapid progress, until we have now attained a pitch of excellence of which we have every reason to be proud, though we are not satisfied. Those who are conversant with the details of the work being carried on at these hospitals know that much more can be done. Is this to be by a continuance of the system or by an upheaval and bringing of these hospitals under a central control? The answer is surely the great watchword which has been such a magnificent cry throughout the four years of struggle ending in the complete victory of our English principles—"Carry on."

Through these years of bitter strife the heroism of our actual fighting troops has been matched by the Medical Services, medical officers and their orderlies, and last but by no means least, by our nurses. The war called for an effort utterly beyond the prevision of all the most far-seeing men, and though the R.A.M.C. as it existed was an admirable kernel to the organisation which had to be provided, it was to the efficiency of the staffs of our hospitals that much of the success of the work was due. The services of the consulting physicians and surgeons, of their assistants and of the general practitioners who had been trained by them, were freely called upon. Thousands of nurses were drawn from the hospital staffs, and every need arising from this terrible four years of bloody struggle was adequately met, and, be it known to all, to the credit of these hospitals their civilian work has been carried on amidst the gravest difficulties as efficiently as ever. There must therefore be something extraordinarily good in our voluntary system and in the organisation of these hospitals, which were able to provide for so heavy a strain on their resources, and yet they "carried on."

### The Success of the Voluntary Hospital.

What is the root of this efficiency? To enable us to form an accurate judgment on this point let us glance for a moment at the typical organisation of a voluntary hospital. First, each hospital depends for its existence on the support of the people. It must have won the confidence of those who contribute to its support. Those who need to resort to the hospital in times of sickness or accident must feel that they, by going to the voluntary hospital, get the best possible treatment, and this treatment must be given not only efficiently but economically, for we may be sure that the voluntary donor will not continue to support any hospital where he does not feel that his gift is put to the best possible use and not wasted.

**Reconstruction and the Voluntary Hospitals—(*cont.*)**

Further, by its work is each hospital known, and as it is known so does it secure the support of its subscribers, and, remember, these subscribers come from every class of the community. Much of the success of these hospitals has been due to the fact that their management has been in the hands of able men, who have freely given their time and their abilities to the supervision of the institutions which have enlisted their services. These men have worked with a whole-hearted selflessness, with one and only one object in view—the amelioration of the lot of those less fortunate than themselves. Not less important than the efforts of the governors of the hospital have been the services of the medical staff, who have generously given their voluntary services to the care of the patients and have taken an enormous pride in the progress of the institutions to which they are attached. *Esprit de corps* has been the stimulus which has retained the services of the distinguished men throughout the country who form the professional staffs of these hospitals. It is but fitting that men who are labouring in their own profession should benefit by the services which they give in the cause of charity. Indeed, they themselves freely admit that their connection with the voluntary hospitals has been the basis of their private practice whereby they live. As teachers working in close co-operation with the hospital managers they aim, like other great teachers in the world's social sphere, at excelling and being known by their work. Each voluntary hospital is striving to hold itself in the forefront in all methods of treatment and in instructing the medical students brought under the influence of its teachers. Indeed, we owe our progress to these conditions, coupled with the fact that the governors have ever been ready to provide at all times the means to promote continuous progress.

Side by side with the education of medical men stands the education of our nurses. The nursing profession of Great Britain is second to none in the world, and in the front rank stand the nursing schools of the voluntary system. When we are considering the nursing profession our thoughts naturally turn to the training of nurses in the greater Poor-Law infirmaries, where invaluable work has been done under the medical superintendents and matrons. But the public control under which they have exercised their functions has stood in their way and failed to leave them free to make the great strides which have characterised the progressive development of the schools attached to our voluntary hospitals.

Let us dwell for one moment on the question of research. Here it must be admitted that the progress made has been to some extent disappointing. The reason is the lack of funds. It appeals to a generous contributor to contribute funds for a hospital to treat the sick, but the necessity for progress in medical knowledge has seldom been brought home to the mind of the average hospital supporter, with the exception of a few notable contributors. What more striking example of this fact than the policy of King Edward's Hospital Fund, the greatest distributor of voluntary hospital benefactions in the world? It has been the policy of this great fund to demand from each hospital an assurance that all the money that they give to it shall go to the treatment of the sick poor, and not to the medical school, where science is taught and progress by research secured.

Do we realise how we suffer in this respect in comparison with the great voluntary hospitals of America, where their rich men have grasped the enormous importance of the endowment of research at the hospitals? Most of our hospitals have no funds for research. Yet during the past thirty years those familiar with voluntary hospital work at home cannot but be amazed at the great progress that has been made in these institutions. The introduction of aseptic surgery, the provision of adequate operating theatres replete with every modern equipment, the thousands of lives which have been saved as a result of the discovery and application of new methods of treatment within our hospitals, wherever they may have originated, have been mainly due to the enterprise of the governors and staffs of our voluntary hospitals. Government has never hesitated to leave to them the task of carrying through any burden of work which may have arisen from the salutary reforms introduced. Without the aid of the voluntary hospitals what would have been the fate of the medical side of the Insurance Act? How could the panel doctor have managed unless he knew that the voluntary hospitals would answer to his call for aid? What would have been the result in regard to tuberculosis? Is the valuable help given in the voluntary hospitals in aiding the maternity section of this great Act appreciated? In recent years it was to the voluntary hospitals that the Local Government Board turned for aid to carry out its important scheme for fighting venereal disease and acknowledges the valuable aid which has been given by them. In still more recent times, what would the Ministry of Pensions have done without the assistance now being given in the voluntary hospitals to the treatment of our discharged maimed soldiers? In treating venereal disease and in caring for war pensioners the hospitals are directly co-operating with the responsible Ministries. Here we have some indication of approaching changes in the future of the voluntary hospitals. The Government has sought the aid of these great institutions and agreed to contribute its share of the cost by making grants-in-aid to the hospitals. That such aid can be rendered on a basis economical to the State has been proved.

### Patients' Interests must Remain Supreme.

There should be no difficulty in organising a Medical Service under which the independence of the voluntary hospitals is preserved and their efforts at progress encouraged. Let us remember that it is the "personal" interest in the patient that has been the characteristic feature of their policy, and much should be sacrificed so that this happy condition should be rigidly preserved. Expenses have increased enormously, whilst there is some justification for fearing that hospital supporters may relax their efforts, so that voluntary hospitals under the old system may be seriously cramped in their good work. It is hoped therefore that the new Ministry of Health will make every effort to develop the best features of our voluntary hospitals and to bring home to the Treasury the necessity of giving adequate financial support free from harassing restrictions wherever the enterprise of individual hospitals justifies such action, whilst it leaves the hospitals at liberty to continue and develop their progressive policy of the past.

The services of the medical profession must be adequately recognised in any arrangements that are made. Give the hospitals their chance, trust them to do the work, retain their independence of administration, and afford them all necessary financial aid.

